# Involvement in bullying and sleep disorders in Chinese early adolescents

**DOI:** 10.3389/fpsyt.2023.1115561

**Published:** 2023-04-27

**Authors:** Han Ding, Leilei Cao, Baoyu Xu, Yuan Li, Jinyu Xie, Jun Wang, Puyu Su, Gengfu Wang

**Affiliations:** ^1^Department of Maternal, Child and Adolescent Health, School of Public Health, Anhui Medical University, Hefei, Anhui, China; ^2^Key Laboratory of Population Health Across Life Cycle, Anhui Medical University, Ministry of Education of the People’s Republic of China, Hefei, Anhui, China; ^3^Anhui Provincial Key Laboratory of Population Health and Aristogenics, Hefei, Anhui, China

**Keywords:** sleep disorder, school bullying, early adolescents, Chinese, cross-sectional

## Abstract

**Background:**

School bullying may cause sleep disorders in early adolescents. Here, we determined the relationship between school bullying (considering all the features of bullying involvement) and sleep disorders, which are the common problems in Chinese early adolescents.

**Materials and methods:**

We conducted a questionnaire survey among 5,724 middle school students from Xuancheng, Hefei, and Huaibei cities in Anhui province, China. The self-report questionnaires included the Olweus Bully/Victim Questionnaire and Pittsburgh Sleep Quality Index. We used latent class analysis to identify the potential subgroups of bullying behavior. Logistic regression analysis was used to investigate the association between school bullying and sleep disorders.

**Results:**

Active participants in bullying interactions, including the bullies and victims, reported higher levels of sleep disorders compared with the non-active participants [Bully: physical (aOR = 2.62), verbal (aOR = 1.73), relational (aOR = 1.80), and cyber (aOR = 2.08); Victim: physical (aOR = 2.42), verbal (aOR = 2.59), relational (aOR = 2.61), and cyber (aOR = 2.81)]. A dose–response relationship was observed between the number of school bullying types and sleep disorders. In the context of bullying roles, bully-victims had the highest risk of reporting sleep disorders (aOR = 3.07, 95% CI: 2.55–3.69). We identified four potential categories of school bullying behaviors: low involvement in bullying, verbal and relational victims, medium bully-victims, and high bully-victims, and the highest frequency of sleep disorders was observed in the high bully-victims group (aOR = 4.12, 95% CI: 2.94–5.76).

**Conclusion:**

Our findings indicate a positive correlation between bullying roles and sleep disorders in early adolescents. Therefore, targeted intervention for sleep disorders should include an evaluation of bullying experiences.

## Introduction

Bullying is a highly prevalent problem among adolescents. The number of children who are regularly bullied in schools ranges from 6 to 46% worldwide (1). Adolescents repeatedly indulge in bullying behavior (2). One study reported a high prevalence of bullying in Chinese adolescents (3). The estimated prevalence of face-to-face bullying victimization and perpetration was 20.8 and 10.3%, respectively, while the estimated prevalence of cyberbullying victimization and perpetration was 9.6 and 8.4%, respectively, in the Chinese school-age population (3). Bullying victimization not only leads to depression, anxiety, and suicide in children but also impacts their educational and economic outcomes (4, 5). In addition, bullying may adversely affect sleep quality (6–9).

Likewise bullying, sleep problems are common occurrence in adolescents. The global prevalence of moderate and severe sleep disorders in adolescents is 28.1% and 8.4%, respectively (10). Recently, a meta-analysis revealed that the prevalence of sleep disorders among middle school students is 20% in Chinese adolescents (11). Sleep disorders may adversely affect on neurodevelopment, cognitive function, mental health, substance use, metabolic function, and academic performance in adolescents (12–19).

Increasing evidence has indicated that bullying is associated with sleep disorders in adolescents; however, the relationship has not been clearly defined. Several features of bullying may influence this association. First, traditional bullying and cyberbullying have variable impacts (20). Cyberbullying occurs in digital environments; therefore, health outcomes might be different (8). Notably, some studies have found that the emotional impact of cyberbullying on victims is more severe than that of traditional bullying (21, 22). Additionally, Chen et al. reported that the risk of sleep disorders was directly proportional to the types of bullying involvement (9). Children who experienced ≥4 victimization types were more likely to have symptoms of sleep disorders and daytime dysfunction than those who experienced 1–3 victimization types and those who were non-victims (9). Second, different roles of involvement in bullying may also have different effects on sleep quality. Bullies and bully-victims are at a higher risk of having poor sleep quality (23). A report suggests that the bully-victims show more subjective sleep disturbances than pure-bully or neutral populations (24). Finally, bullying is a complex behavior and bullying roles and types overlap each other (25). Adolescents involved in bullying may have different experiences as bullies or victims (25). Considering these factors, adolescent involvement in bullying may have different patterns. Individuals involved in bullying exhibit specific patterns such as victim, perpetrator, victim-perpetrator, and neither (26). Of these, victim-perpetrator is associated with subsequent internalization, externalization, and increased risk of school-related problems (27). Therefore, an understanding of different patterns of bullying can elaborate on the relationship between bullying and sleep problems. However, a limited number of studies have systematically examined the correlation between bullying roles and sleep disorders in China. A comprehensive understanding of the association between bullying and sleep disorders could assist in designing targeted intervention strategies for sleep disorders in adolescents.

Here, we focused on sleep patterns and bullying in early adolescents. Early adolescence (10–14 years) is a key development stage, involving crucial neurobiological, hormonal, and socio-contextual changes that influence sleep–wake regulation (28, 29). Early adolescents are also at an elevated risk of bullying and victimization because of their developmental characteristics, which can make them more vulnerable to social dynamics and lead to adverse outcomes such as sleep disorders (30). We hypothesized that engagement in bullying might be associated with sleep disorders in adolescents. Therefore, we formulated the following specific research questions (RQ):

RQ1: Were different types of school bullying associated with sleep disorders?

RQ2: Was involvement in bullying as bully-victims associated with a higher odds ratio for sleep disorders?

RQ3: Were specific patterns of involvement in bullying considering bullying types and roles related to sleep disorders?

## Materials and methods

### Participants

Participants were selected from the Anhui Province in central China using a three-stage random cluster-sampling approach. The protocol has already been detailed in our previous study (31). We invited 5,832 students to participate in this study based on random cluster sampling. The response rate was 98.1%, and 5,724 students (average age: 13.5 ± 1.0 years; 3,006 (52.5%) boys and 2,718 (47.5%) girls) submitted valid questionnaires. The study protocol was approved by the Biomedicine Ethical Committee of Anhui Medical University (Approval no. 20180083), China. Written informed consent was obtained from children and their parents before enrolling them in the study.

### Measures

#### Bullying experiences

Participants were asked to complete the questionnaire for bullying victimization and perpetration within the past 6 months. The questionnaire consisted of 16 items to assess experiences of bullying others and being bullied (physical, verbal, relational, or cyberbullying). The response options for measuring all types of bullying experiences were: 1 = none, 2 = less than twice a month, 3 = 2 to 3 times a month, and 4 = more than once a week. Traditional bullying and cyberbullying were measured using the same standards (bullying two to three times a month or more) to obtain comparable data (32). Internal consistency was also high for each questionnaire, ranging from 0.74 to 0.80 (2). Further, the reliability of the bullying victimization and bullying perpetration was good and the values of Cronbach’s alpha were 0.77 and 0.80, respectively.

#### Sleep quality

We assessed self-reports on sleep quality using the Pittsburgh Sleep Quality Index (PSQI). The index comprised seven dimensions to reflect the sleep status in the last month (1): sleep quality (2), sleep latency (3), sleep duration (4), sleep efficiency (5), sleep disturbances (6), hypnotic drug use, and (7) daytime dysfunction. Each dimension was scored from 0 to 3, and participants with a cumulative score of >7 were considered to have sleep disorders (33). The retest outcomes in 168 students were consistent, ranging from 0.83 to 0.91. The reliability of PSQI was good, and the value of Cronbach’s alpha was 0.78.

#### Covariates

As in our previous studies (2), covariates included sex, grade, family structure, self-perceived family economic status, relationship with both parents, and the number of friends (See Table 1).

**Table 1:** Unadjusted odds ratios (with 95% CI) for sleep disorder (*N* = 5,724).

Category	%	Sleep disorder	
		%	OR (95%CI)
Total	100.0	17.7	
Sex			
Males	52.5	14.2	1.00 [Ref]
Females	47.5	21.7	**1.68 [1.46,1.93]**
Grade			
Grade 7	35.1	13.8	1.00 [Ref]
Grade 8	34.0	19.4	**1.50 [1.27,1.77]**
Grade 9	30.9	20.4	**1.60 [1.35,1.90]**
Family structure			
Nuclear family	56.3	17.3	1.00 [Ref]
Single-parent family	14.5	22.8	**1.41 [1.17,1.70]**
Large family	27.9	15.7	0.89 [0.76,1.05]
Other	1.3	25.7	1.65 [0.97,2.81]
Self-estimated family economic status			
Low	8.7	26.9	1.00 [Ref]
Middle	74.7	17.0	**0.56 [0.45,0.69]**
High	16.6	16.4	**0.53 [0.41,0.69]**
Relationship with mother			
Poor	30.2	26.3	1.00 [Ref]
General	45.0	14.6	**0.48 [0.41,0.56]**
Good	24.9	13.1	**0.42 [0.35,0.51]**
Relationship with father			
Poor	42.5	23.6	1.00 [Ref]
General	39.0	13.5	**0.51 [0.43,0.59]**
Good	18.5	13.4	**0.50 [0.41,0.61]**
Number of friends			
<3	29.2	23.8	1.00 [Ref]
3 ~ 5	40.8	16.1	**0.62 [0.53,0.72]**
≥6	30.0	14.0	**0.52 [0.44,0.62]**

% refers to percent of positive sleep disorder in each demographic category. OR, odds ratios; CI, confidence interval.

Variable levels significant at *p* < 0.05 are in boldface type.

### Statistical analyzes

First, we coded the variables to measure the involvement of participants in bullying, including different types of bullying (perpetration and victimization), the number of these different types, and their role in bullying (please see Supplementary Table S1 for details). Then, we used latent class analysis (LCA) to explain the patterns of involvement considering bullying types and roles (34). Several LCA models were explored using Mplus 7.4 software, where categories were specified from categories 1 to 6. We reported six model fit indexes to determine the best-fit model, namely Akaike Information Criterion (AIC), Bayesian Information Criterion (BIC), adjusted Bayesian Information Criterion (aBIC), Bootstrapped Likelihood Ratio Test (BLRT), Lo–Mendell–Rubin (LMR), and Entropy (34). For AIC, BIC, and aBIC, lower values indicated better model fit. For LMR and BLRT, low and significant *p* values (*p* < 0.05) indicated that the k + 1 class is a better model than the k class. A higher entropy value (>0.6) indicates a better model fit.

SPSS 23.0 (SPSS, Chicago, IL, United States) was used to perform logistics regression analysis and obtain the odds ratios (ORs) and 95% confidence interval (CI) of covariates with sleep disorders. We determined the relationship between school bullying and sleep disorders after adjusting for covariates. The adjusted ORs (aORs) were estimated from logistic regression models after the adjustment of covariates.

## Results

A total of 5,724 adolescents, aged 10–14 years, were included in the study. Approximately 17.7% of the participants reported sleep disorders. Univariate analysis revealed that sex, grade, family structure, self-estimated family economic status, relationship with both parents, and the number of friends correlated with the occurrence of sleep disorders (See Table 1).

### RQ1: Association between bullying types and sleep disorders

Table 2 shows the individual types of school bullying and sleep disorders in adolescents. Physical, verbal, relational, and cyberbullying increased the risk of sleep disorders in bullies, and physical bullying had the greatest impact on the occurrence of sleep disorders (physical: aOR = 2.62, 95% CI: 2.07–3.32; verbal: aOR = 1.73, 95% CI: 1.48–2.03; relational: aOR = 1.80, 95% CI: 1.46–2.20; and cyber: aOR = 2.08, 95% CI: 1.57–2.75). Similarly, all types of bullying increased the risk of sleep disorders in victims of bullying, and cyberbullying had the greatest impact on sleep disorders (physical: aOR = 2.42, 95% CI: 1.93–3.03; verbal: aOR = 2.59, 95% CI: 2.21–3.03; relational: aOR = 2.61, 95% CI: 2.22–3.05; and cyber: aOR = 2.81, 95% CI: 2.16–3.65).

**Table 2:** Multivariate logistic regression between individual type of school bullying and adolescent sleep disorder (*N* = 5,724).

Type of school bullying experience	%	Sleep disorder	
		%	aOR(95%CI)
Bully			
Physical	6.5	32.9	**2.62 [2.07,3.32]**
Verbal	23.0	23.7	**1.73 [1.48,2.03]**
Relational	9.9	27.6	**1.80 [1.46,2.20]**
Cyber	4.5	31.2	**2.08 [1.57,2.75]**
Victim			
Physical	7.6	32.1	**2.42 [1.93,3.03]**
Verbal	20.1	30.4	**2.59 [2.21,3.03]**
Relational	17.9	32.5	**2.61 [2.22,3.05]**
Cyber	4.9	36.7	**2.81 [2.16,3.65]**

% refers to percent of positive sleep disorder in each type of school bullying experience.

Different types of bullying perpetration and victimization were separately put into the model for estimation. In addition, participants who did not have such bullying and victimization were considered a reference group.

Model adjusted for gender, grade, family structure, self-estimated family economic status, relationship with mother, relationship with father and number of friends that were statistically significant in univariate analyzes.

aOR, adjusted odds ratios; CI, confidence interval.

Results arrived statistically significant at *p* < 0.05 are in boldface type.

Participants who were involved as bullies were more prone to report sleep disorders than those who did not engage in school bullying (four types: aOR = 3.83, 95% CI: 2.20–6.68; See Table 3). Similarly, victims of all types of bullying had significantly higher OR values for sleep disorders (four types: aOR = 5.57, 95% CI: 3.71–8.37). An increase in the number of bullying types significantly increased the risk of sleep disorders among individuals who bullied others or were victimized (bully: aOR = 1.45, 95% CI: 1.34–1.57 and victim: aOR = 1.61, 95% CI: 1.51–1.72).

**Table 3:** Multivariate logistic regression of adolescent sleep disorder on number of school bullying types (*N* = 5,724).

Variety of school bullying experience	%	Sleep disorder	
		%	aOR(95%CI)
Bully			
Non-involved	70.6	15.0	1.00 [Ref]
One type	19.3	21.1	**1.52 [1.27,1.81]**
Two types	6.6	28.3	**2.34 [1.82,2.99]**
Three types	2.5	31.3	**2.80 [1.92,4.07]**
Four types	1.0	40.4	**3.83 [2.20,6.68]**
Number of types			**1.45 [1.34, 1.57]**
Victim			
Non-involved	70.0	13.1	1.00 [Ref]
One type	16.9	23.6	**1.98 [1.66,2.37]**
Two types	7.4	32.2	**3.04 [2.41,3.83]**
Three types	3.8	36.3	**3.77 [2.79,5.11]**
Four types	1.9	44.3	**5.57 [3.71,8.37]**
Number of types			**1.61 [1.51,1.72]**

% refers to percent of positive sleep disorder in each type of school bullying experience.

The number of perpetration and victimization of bullying behaviors were separately entered into the model.

Model adjusted for gender, grade, family structure, self-estimated family economic status, relationship with mother, relationship with father and number of friends that were statistically significant in univariate analyzes.

aOR, adjusted odds ratios; CI, confidence interval.

Results arrived statistically significant at *p* < 0.05 are in boldface type.

### RQ2: Role In bullying and sleep disorders

We categorized the participants into three groups according to their roles in school bullying, namely, bully only, victim only, and bully-victim. All three subgroups had a higher risk of sleep disorders compared to those who had no bullying experience, and the bully-victim group had the highest prevalence of sleep disorders (bully only: aOR = 1.69, 95% CI: 1.35–2.10; victim only: aOR = 2.71, 95% CI: 2.24–3.29; and bully-victim: aOR = 3.07, 95% CI: 2.55–3.69; See Table 4).

**Table 4:** Multivariate logistic regression of adolescent sleep disorder on role in school bullying (*N* = 5,724).

Role of school bullying	%	Sleep disorder	
		%	aOR(95%CI)
Non-involved	56.7	12.0	1.00 [Ref]
Victim only	13.9	27.7	**2.71 [2.24,3.29]**
Bully only	13.3	18.0	**1.69 [1.35,2.10]**
Bully-victim	16.1	29.5	**3.07 [2.55,3.69]**

% refers to percent of positive sleep disorder in each type of school bullying experience.

Model adjusted for gender, grade, family structure, self-estimated family economic status, relationship with mother, relationship with father and number of friends that were statistically significant in univariate analyzes.

aOR, adjusted odds ratios; CI, confidence interval.

Variable levels significant at *p* < 0.05 are in boldface type.

### RQ3: Patterns of involvement In bullying and sleep disorders

LCA was conducted for up to six classes of tests. We identified four latent classes, and the model-fitting statistics are shown in Supplementary Table S2. We classified bullying patterns into four categories: low involvement in bullying, verbal and relational victims, medium bully-victims, and high bully-victims.

Table 5 shows the relationship between the pattern of school bullying and sleep disorders. Compared with those who had low involvement in bullying, verbal and relational victims, medium bully-victims, and high bully-victims had significantly higher ORs for sleep disorders (verbal and relational victims: aOR = 3.26, 95% CI: 2.59–4.10; medium bully-victims: aOR = 2.33, 95% CI: 1.94–2.80; and high bully-victims: aOR = 4.12, 95% CI: 2.94–5.76).

**Table 5:** Multivariate logistic regression between pattern of school bullying and adolescent sleep disorder (*N* = 5,724).

Pattern of school bullying	%	Sleep disorder	
		%	aOR(95%CI)
Low involvement in bullying	75.8	13.7	1.00 [Ref]
Verbal and relational victims	7.2	34.6	**3.26 [2.59,4.10]**
Medium bully-victims	14.1	26.6	**2.33 [1.94,2.80]**
High bully-victims	2.9	38.3	**4.12 [2.94,5.76]**

% refers to percent of positive sleep disorder in each pattern of school bullying experience.

Model adjusted for gender, grade, family structure, self-estimated family economic status, relationship with mother, relationship with father and number of friends that were statistically significant in univariate analyzes.

aOR, adjusted odds ratios; CI, confidence interval.

Variable levels significant at *p* < 0.05 are in boldface type.

## Discussion

We explored the relationship between school bullying and sleep disorders in early adolescents considering distinct features of bullying, including types, numbers, roles, and patterns. We found that both traditional and cyberbullying were significantly associated with sleep disorders, which is consistent with the previous findings (35, 36). The participants who were physically bullied were most likely to report sleep disorders. This may be because they received tactile, visual, and other stimuli simultaneously, which left a deeper impression on them (37). We also found that victims of cyberbullying had the highest risk of sleep disorders. Cyberbullying can occur at any time, even when the victim is not physically present. Moreover, its anonymity may increase the psychological vulnerability of the victim leading to severe adverse effects (38). The effects of cyberbullying are pervasive and long-lasting for victims, and they may have a harder time coping with the adverse effects than victims of physical bullying (39). Notably, for all four bullying types, the aORs for sleep disorders in the victims were higher than that of the bullies, suggesting that the sleep problems experienced by the victims were more severe than those experienced by bullies. The experience of school bullying may cause traumatic stress in adolescents, which can have significant physiological and psychological effects (40). The cognitive activation theory of stress (41) states that cognitive activation is a key factor in physiological arousal and stress response. In the context of bullying and sleep disturbance, bullying triggers physiological arousal, which can interfere with sleep patterns. Further, bullying may lead to dysfunction of the hypothalamic–pituitary–adrenal axis, thereby increasing the circulating cortisol levels. The elevated cortisol increases physiological arousal, thereby disrupting normal sleep rhythms (42). Peer victimization predicted increased inflammatory responses to social stressors in vulnerable adults (43), and higher levels of C-reactive protein were associated with sleep disturbances (44). Taken together, cyberbullying was a serious issue for adolescents, and victims were at a higher risk of sleep disturbances than bullies (See Table 2).

Second, we found that a dose–response relationship was observed between the number of bullying types (perpetration and victimization) and sleep disorders. Several researchers have shown a cumulative association between participation in more bullying types and mental health outcomes such as depression and anxiety (45, 46). Consistent with our results, a previous study also reported that involvement in multiple bullying types results in a higher risk of sleep disorders (7). Participating in multiple types of bullying may have a greater negative effect on sleep disorders than participating in a single type of bullying. This happens because rumination is a common response to bullying, which interferes with sleep (7). Biologically, a dose–response association was observed between the number of times being bullied and CRP levels (43). In addition, a longitudinal study found an association between hair cortisol concentration at the age of 17 and the severity of peer victimization during childhood (47). Therefore, it is essential to pay more attention to early adolescents who are often bullied and provide timely interventions to prevent adverse consequences.

Third, our results showed that all school bullies and victims of bullying had a higher prevalence of sleep disorders, and the aOR for this association was the highest among bully-victims. A Japanese study revealed that intrinsic and extrinsic problems were more frequent among bully-victims in early adolescence (48). Bully-victims, who face the negative effects of both bullying and victimization, have higher Athens Insomnia Scale scores and are at a greater risk for anxiety, depression, and aggressive behavior compared to bullies and victims, thereby increasing their odds of suffering and distress (30). Some authors have concluded that the victims are highly likely to participate in bullying behaviors and that the bully-victim pattern becomes more stable over time, causing more mental health problems in bully-victims than in bullies and victims (49). The “trauma theory” suggests that bully-victims are more likely to experience trauma in their lives and trauma-induced over-activation of a part of the brain produces more reactivity and may have an impact on sleep (50). Overall, our results suggest that bully-victims should be prioritized and urgent interventions should be provided to mitigate the negative consequences.

**Figure 1:**
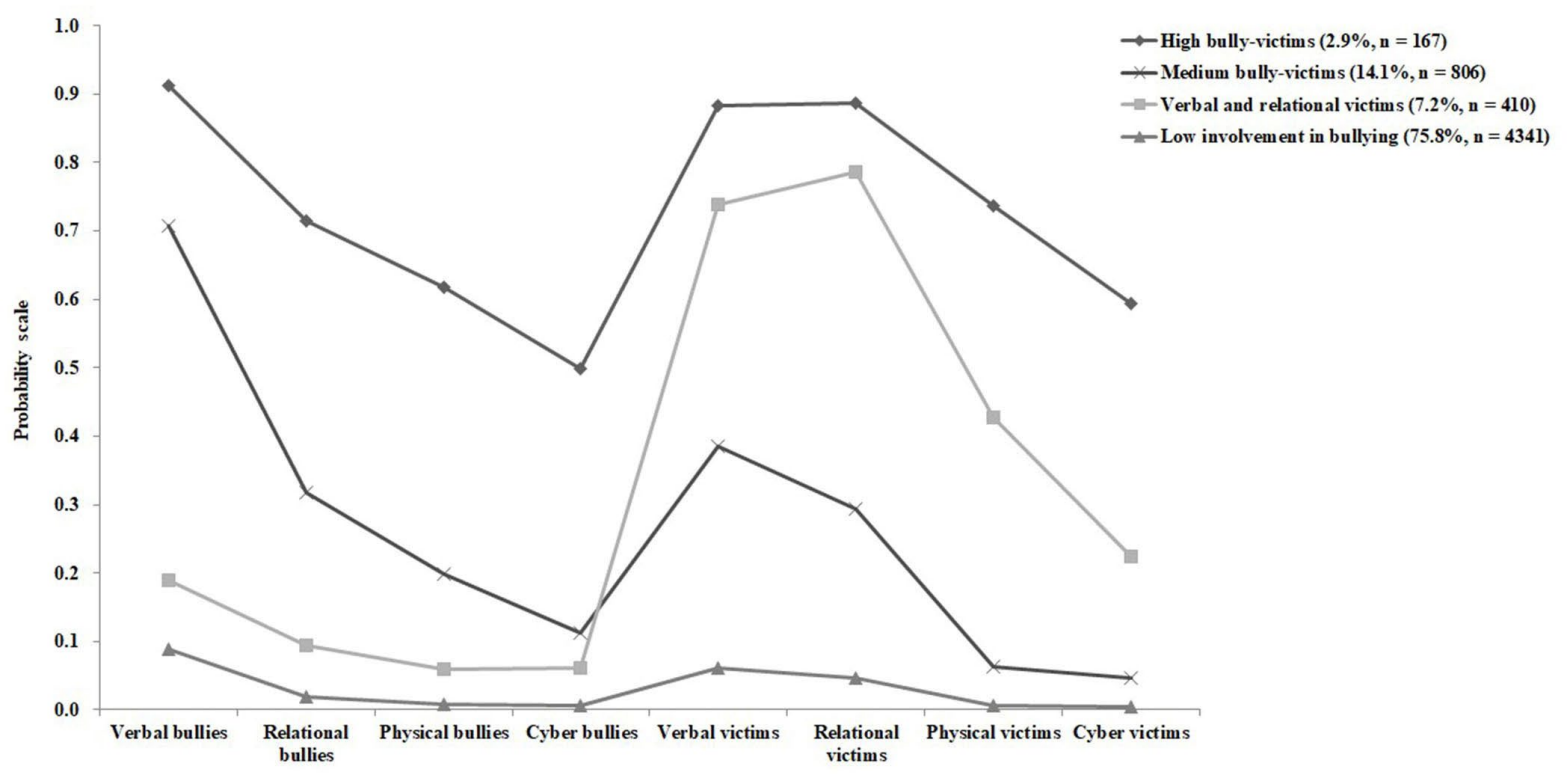
Probability of involvement in school bullying for a four-classes model.

The LCA is a person-centered approach that allows participants to be divided into different potential groups using several key variables or indicators (51), which can help researchers to better understand the complexity of bullying. We identified four different school bullying phenotypes through LCA, including participants with low involvement in school bullying (75.8%), verbal and relational victims (7.2%), medium bully-victims (14.1%), and high bully-victims (2.9%). Although our labels for the categories were different, the results were similar to a previous study reporting LCA-based phenotypes. The highest number of participants was concentrated in the low-bullying group (75.4%) and the lowest numbers were observed in the high-bullying victimization and perpetration group (2.0%) (52). The results showed that the participants in the high bully-victims group had the highest frequency of sleep disorders. A previous study that classified patterns of involvement in bullying using LCA reported that adolescents in the bully-victim group had a higher risk of cocaine use than those in other groups (53). Furthermore, we found a high percentage of verbal and relational bullying among those involved in bullying, and the aOR values were higher in the verbal and relational victims than in the medium bully-victims. Some researchers demonstrated that verbal and relational victimizations were common and associated with more serious health problems than physical victimization (35, 47, 54). Therefore, we need to focus on the victims of verbal and relational bullying in addition to the bully-victims.

Our findings have two important implications. First, we observed a positive relationship between bullying and sleep disorders in early adolescents, and this relationship varies with different bullying types. The prevention of sleep disorders in adolescents should involve a detailed evaluation of bullying experiences considering the types, roles, and patterns of bullying. Second, different bullying types were associated with variable risks of sleep disorders, which suggests that a comprehensive assessment of bullying experiences is necessary to understand the health impacts of bullying on adolescents in future studies. Particularly, given the complexity of school bullying and the overlapping types and roles involved in bullying, it is important to identify potential categories based on a range of behaviors and characteristics, and, in turn, understand the different health impacts caused by different types of bullying and to identify victims who need intervention.

## Limitations

Our research design has some limitations. First, cross-sectional studies cannot prove a causal relationship between bullying in schools and sleep disorders; only a correlation was found between bullying in schools and sleep disorders. Longitudinal methods could be used in future studies to confirm the prospective relationship between school bullying and sleep disorders. Second, our data were biased by the recalling. Especially, the measuring time frame of bullying experiences was set as past 6 months to increase reporting rates. This includes the fact that adolescent bullying tends to be repeated and remains fairly constant over time (55). In addition, information on bullying was collected from a retrospective self-reporting questionnaire and may be influenced by social approval effects that may lead to an underestimation of the level of bullying. Moreover, participants may have underreported their sleep disorders. A method that combines register-based and self-reports can be used in future studies to obtain more reliable data. Third, we included three middle schools in Anhui province, and our results represent a specific set of participants.

## Conclusion

Early adolescents who were involved in bullying had a higher risk of suffering from sleep disorders. A dose–response correlation was observed between the number and frequency of different bullying types and sleep disorders. The role of the adolescent in bullying and the pattern of involvement can also influence the occurrence of sleep disorders. Therefore, parents, schools, and society should be more aware of the detrimental effects of school bullying on sleep patterns in adolescents. This urgent issue demands considerable intervention and new strategies should be implemented to stop bullying in schools for the prevention of sleep disorders in early adolescents.

## Data availability statement

The original contributions presented in the study are included in the article/Supplementary material, further inquiries can be directed to the corresponding authors.

## Ethics statement

The studies involving human participants were reviewed and approved by the Biomedicine Ethical Committee of Anhui Medical University (Approval no. 20180083), China. Written informed consent to participate in this study was provided by the participants’ legal guardian/next of kin.

## Author contributions

HD, PS, and GW contributed to conception and design of the study. HD performed data analyzes, and drafted the manuscript. LC helped with data collection, performed data analysis, and drafted the manuscript. YL helped with the statistical analysis. BX, JX, and JW wrote sections of the manuscript. All authors contributed to manuscript revision, read, and approved the submitted version.

## Funding

This study was funded by the grants from the National Natural Science Foundation of China (Grant No. 82204071, 81874268, and 82173539), grant of the Scientific Research of BSKY from Anhui Medical University (0303033201).

## Conflict of interest

The authors declare that the research was conducted in the absence of any commercial or financial relationships that could be construed as a potential conflict of interest.

## Publisher’s note

All claims expressed in this article are solely those of the authors and do not necessarily represent those of their affiliated organizations, or those of the publisher, the editors and the reviewers. Any product that may be evaluated in this article, or claim that may be made by its manufacturer, is not guaranteed or endorsed by the publisher.
