# Supplementary material for: Involvement in bullying and sleep disorders in Chinese early adolescents
Source: Front Psychiatry. 2023 Apr 27;14:1115561. doi: 10.3389/fpsyt.2023.1115561 (PMC10172573; doi:10.3389/fpsyt.2023.1115561)
Supplement: Supplementary file 1 [file Table_1.docx]

Supplementary Material

**Involvement in bullying and sleep disorders in Chinese Early Adolescents**

**Han Ding ^a,†^, Leilei Cao ^a,†^, Baoyu Xu ^a^, Yuan Li ^a^, Jinyu Xie ^a^, Jun Wang ^a^, Puyu Su ^a,b,c,⁎^, Gengfu Wang ^a,b,c,⁎^**

*** Correspondence:** supuyu@ahmu.edu.cn (P. Su); wanggenfu@ahmu.edu.cn (G. Wang).

**Supplemental Table 1.** Bullying questionnaires in this study.

| Variables | Items |
| --- | --- |
| Physical bullying victimization | In the past six months, have you been bullied at school by the following means? (1) been hit, kicked, pushed, shoved around, or locked indoors; (2) been blackmailed money or damaged things; |
| Verbal bullying victimization | In the past six months, have you been bullied at school by the following means? (1) been called mean names or made fun of or teased in a hurtful way; (2) been called mean names about accent; |
| Relational bullying victimization | In the past six months, have you been bullied at school by the following means? (1) been excluded by others from their group of friends or been leaved out of things on purpose; (2) others telling lies or spreading false rumors about me or sending mean notes and trying to make me unpopular; |
| Cyber bullying victimization | In the past six months, have you been bullied at school by the following means? (1) been bullied using a computer or email messages or pictures; (2) been bullied using a cell phone. |
| Physical bullying perpetration | In the past six months, have you bullied others at school by the following means? (1) hitting, kicking, pushing, shoving around, or locking someone indoors; (2) blackmailing someone for money or damaging things; |
| Verbal bullying perpetration | In the past six months, have you bullied others at school by the following means? (1) calling someone mean names or making fun of or teasing someone in a hurtful way; (2) calling someone mean names about accent; |
| Relational bullying perpetration | In the past six months, have you bullied others at school by the following means? (1) excluding others from my group of friends or leaving others out of things on purpose; (2) telling lies or spreading false rumors about others or sending mean notes and trying to make others unpopular; |
| Cyber bullying perpetration | In the past six months, have you bullied others at school by the following means? (1) bullying others using a computer or email messages or pictures; (2) bullying others using a cell phone. |

Note: For all types of bullying experiences, the response options were ‘1=none, 2 = less than twice a month, 3 = 2 to 3 times a month, 4 = more than once a week’.

Firstly, four types of bullying perpetration and victimization was dichotomized using the criterion two to three times a month or more. Then, bullying perpetration experiences were coded as an ordinary variable, i.e., the number of bullying types one perpetrated (i.e., not involved, one type, two types, three types, or four types). Similarly, the number of bullying victimization types one experienced were calculated (i.e., not involved, one type, two types, three types, or four types). Moreover, the students were divided into four groups (not involved, bully only, victim only, and bully-victim) based on their bullying experiences, which has described in our previous study [1].

[1] Wang GF, Jiang L, Wang LH, Hu GY, Fang Y, Yuan SS, Wang XX, Su PY. Examining childhood maltreatment and school bullying among adolescents: a cross-sectional study from Anhui Province in China. J Interpers Violence. 2019;34(5):980–99.

**Supplemental Table 2.** Fitting indexes of different classes of latent class models.

| Model | K | Log | AIC | BIC | *a*BIC | Entropy | LMR | BLRT | Latent class probabilities |
| --- | --- | --- | --- | --- | --- | --- | --- | --- | --- |
| 1 | 8 | -15587.914 | 31191.829 | 31245.048 | 31219.627 |  |  |  |  |
| 2 | 17 | -13648.755 | 27331.509 | 27444.600 | 27390.579 | 0.791 | <0.001 | <0.001 | 0.80031/ 0.19969 |
| 3 | 26 | -13452.506 | 26957.011 | 27129.974 | 27047.354 | 0.713 | <0.01 | <0.001 | 0.22781/ 0.04892/ 0.72327 |
| **4** | **35** | **-13303.193** | **26676.386** | **26909.221** | **26798.001** | **0.774** | **<0.001** | **<0.001** | **0.02918/ 0.07163/ 0.75839/ 0.14081** |
| 5 | 44 | -13251.603 | 26591.205 | 26883.912 | 26744.093 | 0.794 | 0.059 | <0.001 | 0.03145/ 0.75839/ 0.01730/ 0.06813/ 0.12474 |
| 6 | 53 | -13222.074 | 26550.147 | 26902.726 | 26734.307 | 0.824 | 0.0853 | <0.001 | 0.04927/ 0.03372/ 0.06796/ 0.07512/ 0.75891/ 0.01502 |

*Note*. K: Number of Free Parameters; Log: Loglikelihood value; AIC: Akaike Information Criterion; BIC: Bayesian Information Criterion; *a*BIC: the sample-size Adjusted BIC; LMR: Lo-Mendell- Rubin (LMR) adjusted likelihood ratio test; BLRT: Bootstrap Likelihood Ratio Text.
